# Effects of *Litsea cubeba* essential oil on growth performance, blood antioxidation, immune function, apparent digestibility of nutrients, and fecal microflora of pigs

**DOI:** 10.3389/fphar.2023.1166022

**Published:** 2023-07-03

**Authors:** Fengming Chen, Yushi Wang, Kaijun Wang, Jiayi Chen, Ke Jin, Kaiqiang Peng, Xu Chen, Zhimou Liu, Jiang Ouyang, Yong Wang, Xiaoya Zhang, Haowei Zou, Jun Zhou, Binsheng He, Qian Lin

**Affiliations:** ^1^ Hunan Provincial Key Laboratory of the TCM Agricultural Biogenomics, Changsha Medical University, Changsha, Hunan, China; ^2^ Institute of Bast Fiber Crops, Chinese Academy of Agricultural Sciences, Changsha, China; ^3^ State Key Laboratory for Conservation and Utilization of Subtropical Agro-bioresources, College of Animal Science and Technology, Guangxi University, Nanning, Guangxi, China; ^4^ Hunan Nuoz Biological Technology Co., Ltd., Yiyang, Hunan, China; ^5^ College of Animal Science and Technology, Hunan Agricultural University, Changsha, Hunan, China

**Keywords:** essential oil, antioxidant, microbiota, gut, pig

## Abstract

The purpose of this experiment was to investigate the effects of *Litsea cubeba* essential oil (LCO) on the growth performance, blood antioxidation, immune function, apparent digestibility of nutrients, and fecal microflora in fattening pigs. A total of 120 pigs were randomly assigned to five groups, with six replicate pens per treatment and four pigs per pen, and they were fed basal diet, chlortetracycline (CTC), and low-, medium-, and high-concentration LCO. The results of the study showed that compared with the control treatment and CTC addition treatment of the basic diet, the catalase level in the serum of the pigs treated with 500 mg/kg LCO in the diet of finishing pigs was significantly increased (*p* < 0.05). The apparent digestibility of crude protein, crude ash, and calcium in pigs with different levels of LCO was significantly increased compared with the control treatments fed the basal diet (*p* < 0.05). In addition, compared with the control treatment fed the basal diet and the treatment with *CTC*, the apparent digestibility of ether extract in pigs treated with medium-dose LCO was significantly increased (*p* < 0.05), and the apparent digestibility of pigs was significantly increased after the addition of low-dose LCO (*p* < 0.05). Among the genera, the percentage abundance of *SMB53* (*p <* 0.05) was decreased in the feces of the *CTC* group when compared to that in the medium-LCO group. At the same time, the relative abundance of *L7A_E11* was markedly decreased in the feces of the control and medium- and high-concentration LCO group than that in the *CTC* group (*p <* 0.05). In conclusion, adding the level of 250 mg/kg LCO in the diet of pig could improve the growth performance and blood physiological and biochemical indicators of pigs, improve the antioxidant level of body and the efficiency of digestion and absorption of nutrients, and show the potential to replace *CTC*.

## 1 Introduction

Aromatic and volatile oils are obtained from plant tissues by steam distillation. They consist of aliphatic, aromatic, and terpene compounds (Hyldgaard et al., 2012). Essential oils have numerous applications, including spices, food, the chemical industry, and cosmetics. They have certain inhibitory effects on microorganisms (Burt, 2004), making them widely used in these industries. Through research, scholars found that probiotics, plant essential oil, organic acids, etc., as functional additives, can improve animal growth performance, improve animal intestinal health, and have effects similar to that of antibiotics, which is of great significance to the livestock and poultry breeding industry (Suiryanrayna and Ramana, 2015; Liu et al., 2018). At present, the substitutes for antibiotics in feed mainly include organic acids (He RX. et al., 2020), enzyme preparations (Zhang et al., 2020; Vangroenweghe et al., 2021), probiotics (Pan et al., 2017; Csernus and Czeglédi, 2020), antimicrobial peptides (Wang et al., 2016), medium-chain fatty acids (Ren et al., 2020), and essential oils (Li P. et al., 2012; Cheng et al., 2018; Yi et al., 2018). As one of the main antibiotic substitutes, essential oil promotes the growth of weaned piglets and regulates intestinal microorganisms. Plant essential oils are considered potential substitutes for antibiotics and play an active role in improving animal growth performance and preventing diarrhea (Tian and Piao, 2019).

Plant essential oils have antibacterial, anti-inflammatory and antioxidant functions, which can improve the immunity and antioxidation of animals, regulate the structure of intestinal flora, improve intestinal health, and promote animal growth (Zeng et al., 2015a; Zhai et al., 2018). Under normal circumstances, the free radicals produced by the body in the process of oxidative metabolism can be removed by the antioxidant system in time to maintain the balance between oxidation and antioxidation in the body. When the animal body is subjected to stress (such as weaning and bacterial or viral infection), a large number of free radicals are produced in the body, which leads to the imbalance of redox state and oxidative damage (Jiang et al., 2017). A large number of studies have found that essential oils derived from plants (such as oregano essential oil, thyme essential oil, and clove essential oil) have strong antioxidant activity (Bozin et al., 2006; Bounatirou et al., 2007; Wei and Shibamoto, 2010; Teixeira et al., 2013). In addition, phenols, aldehydes, and their derivatives in the main components of essential oil have antioxidant function (Amorati et al., 2013). Essential oils can exert their antioxidant function in two ways: 1) essential oils can directly react with oxygen free radicals, thus reducing the number of oxygen free radicals in the body (Foti, 2007); 2) essential oils can regulate the activities of antioxidant enzymes in the body and indirectly participate in antioxidant function (Choi et al., 2020).

Essential oil also has the function of promoting the growth of intestinal probiotics and protecting against pathogenic bacteria and inhibiting their proliferation, thus improving the structure of intestinal flora (Ouwehand et al., 2010). The mechanism of essential oil inhibiting pathogenic bacteria may be that its active components have strong surface activity and fat solubility, can quickly penetrate the cell membrane of pathogenic microorganisms, make their contents flow out, and effectively prevent the process of respiratory oxidation in mitochondria. Microorganisms lose their energy source and die (Di Pasqua et al., 2007). *L. cubeba* is a deciduous shrub or small tree with high appreciation value and application value in China. *L. cubeba* essential oil (LCO) is composed of *trans-citral*, *cis-citral*, *d-limonene*, and other chemicals derived from the fruit of *L. cubeba* (Thielmann and Muranyi, 2019). LCO is active against *V. parahaemolyticus*, *L. monocytogenes*, *H. anomala* (Liu and Yang, 2012), and *Botrytis cinerea*, a fungus causing the putrefaction of fruits and vegetables (Wang L. et al., 2019). The purpose of this experiment is to explore the effects of LCO on growth performance, apparent digestibility of nutrients, serum immunity, and antioxidant indexes of Taoyuan black pigs, so as to provide data support for the rational application of LCO in pigs.

## 2 Materials and methods

### 2.1 Ethical approval

All the experiment procedures were reviewed and approved by the animal care committee of Changsha Medical University (No. 2021-08), Changsha, China.

### 2.2 Animals, diets, and treatments

The experiment adopted a single-factor randomized trial design. The 120 fattening pigs of the same breed with good health status and similar body weight were selected and randomly divided into five treatments (six replicates per treatment and four pigs per replicate). In addition, the animals used in the experiment were prefed for 7 days, and the formal test was conducted for 35 days. The test groupings are shown in Table 1.

**TABLE 1:** Experimental design and grouping.

Group	Diet
Con	Basal diet
CTC	Basal diet +75 g/t chlortetracycline (15%) premix
LCO1	Basal diet +250 g/t LCO
LCO2	Basal diet +500 g/t LCO
LCO3	Basal diet +1000 g/t LCO

The basal diet used in the experiment was formulated with reference to the nutritional requirements of fattening pigs in NRC (2012) and Chinese pig breeding standards (NY-T65-2004). The diet was composed of corn, soybean meal, and rice bran meal. The nutrition level is shown in Table 2, and the test pigs were fed pelleted feed for the whole period.

**TABLE 2:** Composition and nutrition levels of the basal diet (air-dry basis, %).

Item	Content
Ingredients	
Corn	27.50
Rice bran	10.00
Rice bran meal	16.00
Broken rice	30.00
Soybean meal	12.50
Premix1)	4.00
Total	100.00
Nutrient level2	
DE (Mcal/kg)	3.20
CP	15.02
Ca	0.55
TP	0.76
AP	0.20
Lys	1.15
Thr	0.60
Met	0.25
Met + Cys	0.50

1) Premix contained per kg VA, 325IU; VD, 37.5IU; VE, 2.75IU; VK_3_, 0.013 mg; VB_2_, 0.63 mg; VB_6_, 0.25 mg; VB_12_, 2.5 mg; biotin, 0.013 mg; folic acid, 0.08 mg; D-pantothenic acid, 2.00 mg; hydrochloric acid, 2.5 mg; choline chloride, 0.08 mg; antioxidants, 12.50 mg; FeSO_4_·H_2_O, 12.50 mg; CuSO_4_·H_2_O, 0.88 mg; ZnO, 15.00 mg; MnSO_4_·H_2_O, 0.50 mg; Na_2_SeO_3_ 0.04 mg; KI, 0.04 mg. 2) Digestible energy was a calculated value.

### 2.3 Sampling and collection

At the beginning and end of the experiment, the test pigs were weighed on an empty stomach in repeated units. During the test period, the daily feed intake of each repeated test pig was recorded, and the average daily feed intake (ADFI), average daily gain (ADG) and feed-to-weight ratio (F/G) of the test pigs were calculated. (ADG) and feed-to-weight ratio (F/G). We used disposable sterile gloves to collect 500 g of feed samples for each treatment, and at the same time, we randomly collected fresh and clean fecal samples in each replicate, approximately 1 kg for each replicate. Then, we stored them in separate packages for testing.

For each repetition, 20 mL of blood was collected from the anterior vena cava from a pig close to the average body weight of the treatment, placed in an ordinary centrifuge tube, kept at room temperature for 30 min, centrifuged at 3500 r/min for 10 min, and separated. Serum was divided into 1.5 mL centrifuge tubes and stored at −80 C for routine physiological, biochemical, antioxidant, and immune indicators of the serum to be tested.

### 2.4 Serum index detection

The serum levels of aspartate aminotransferase (AST), alanine aminotransferase (ALT), alkaline phosphatase (ALP), urea, glucose (GLU), total cholesterol (TC), triglyceride (TG), low-density lipoprotein (LDL), high-density lipoprotein (HDL), total protein (TP), and albumin (ALB) in the pigs were measured by using an automatic biochemical analyzer, and the level of globulin (GLB) was calculated (Wang et al., 2022a). A spectrophotometer or microplate reader was used to test total antioxidant capacity (T-AOC), superoxide dismutase (SOD), glutathione (GSH), glutathione peroxidase in pig serum (GPX), catalase (CAT), and malondialdehyde (MDA) levels, including the determination of six antioxidant indicators. The levels of IgA, IgG, and IgM and the levels of complements C3 and C4 in the serum of the test pigs were determined by the enzyme-linked immunosorbent assay.

### 2.5 Nutrient digestibility determination

The digestibility of dietary dry matter, energy, crude protein, crude fat, crude fiber, crude ash, calcium, and phosphorus was analyzed by the endogenous indicator (acid-insoluble ash) method.

### 2.6 DNA extraction and PCR amplification

As previously reported, DNA extraction of fecal samples and 16S ribosomal RNA amplification were carried out (Wang et al., 2022b). Fecal samples were extracted for DNA using an E.Z.N.A. ^®^ Soil DNA Kit (Omega Biotek, Norcross, GA, United States) on the basis of the standard protocol. Using universal primers targeting the V3–V4 region 338F/806R, 16S rRNA from bacteria was amplified and sample sequenced using an Illumina Miseq PE300 platform (Illumina, SD, United States) (Wang H. et al., 2019). Sequence reads from the original sequence were uploaded to NCBI’s Sequence Read Archive under accession number PRJNA953808.

### 2.7 Statistical analysis

After preliminary processing of the experimental data with Excel 2007 software, one-way ANOVA was performed with SPSS 19.0 statistical software. If the difference between groups was significant (*p* < 0.05), Duncan’s method was used for multiple comparisons. Furthermore, 0.05 < *p* < 0.1 was considered a trend. Test results are presented in the form of mean ± standard deviation. In addition, orthogonal polynomial contrasts were used to analyze the linear and quadratic effects of different dietary levels of LCO on various indicators of pigs.

## 3 Results

### 3.1 Effect of dietary LCO levels on growth performance in Taoyuan black pigs

The effects of different dietary levels of LCO on the growth performance of finishing pigs are shown in Table 3. Compared with the control treatment of the basal diet and the addition of chlortetracycline, the addition of different levels of LCO in the finishing pig diet had no effect on the average final weight, ADG, and ADFI of the test pigs (*p* > 0.05). However, from the numerical point of view, the average final weight and ADG of the pigs in the experimental treatment with 250 mg/kg of LCO in the diet were the highest, and the diets supplemented with different levels of LCO improved the ADFI compared with the control group of the basal diet. At the same time, dietary supplementation with different levels of LCO had a tendency to reduce the feed-to-weight ratio of pigs (*p* = 0.078), and the treatment group supplemented with 250 mg/kg LCO in the diet obtained the best feed-to-weight ratio.

**TABLE 3:** Effect of dietary LCO levels on growth performance in *Taoyuan* black pigs (kg).

Item	CTC level, mg/kg	LCO levels, mg/kg				SEM	*p*-value	*p*-value	
	75	0	250	500	1000			Linear	Quadratic
Average initial weight (kg)	82.03 ± 2.46	82.69 ± 2.84	80.83 ± 4.68	80.97 ± 3.26	81.67 ± 4.30	0.624	0.894	0.578	0.463
Average final weight (kg)	107.63 ± 2.91	107.36 ± 3.32	109.47 ± 4.39	108.58 ± 3.86	107.36 ± 5.30	0.704	0.866	0.941	0.327
ADG (kg)	0.74 ± 0.06	0.72 ± 0.06	0.83 ± 0.10	0.80 ± 0.10	0.75 ± 0.07	0.015	0.118	0.404	0.023
ADFI (kg)	2.62 ± 0.09	2.50 ± 0.16	2.59 ± 0.10	2.63 ± 0.08	2.63 ± 0.07	0.032	0.191	0.033	0.477
F/G	3.55 ± 0.25	3.50 ± 0.09	3.15 ± 0.28	3.32 ± 0.36	3.55 ± 0.33	0.059	0.078	0.897	0.014

In the same row, values with different small letter superscripts mean a significant difference (*p* < 0.05), the same as in the following.

### 3.2 Effect of dietary LCO levels on serum biochemical indices in Taoyuan black pigs

The effects of different LCO levels in diet on serum physiological and biochemical indexes of finishing pigs are shown in Table 4. Compared with the control treatment and *CTC* addition treatment in the basal diet, adding different levels of LCO to the diet of finishing pigs had no significant effect on the serum GLU, TG, TCHO, ALB, H-DLC, ALP, AST, and ALT levels of pigs (*p* > 0.05). At the same time, the addition of high-level LCO to the diet had a tendency to increase the serum TP and GLB levels of pigs (0.05 < *p* < 0.10), and there was a significant linear positive correlation between this trend and the addition of different levels of LCO to the diet (*p* < 0.05). In addition, compared with *CTC* treatment, the serum L-DLC levels of pigs in the treatment group supplemented with 250 and 500 mg/kg LCO were significantly decreased (*p* < 0.05).

**TABLE 4:** Effect of dietary LCO levels on serum biochemical indices in *Taoyuan* black pigs.

Item	CTC level, mg/kg	LCO levels, mg/kg				SEM	*p*-value	*p*-value	
	75	0	250	500	1000			Linear	Quadratic
GLU/(mmol/L)	2.68 ± 0.67	2.46 ± 0.88	3.62 ± 2.89	2.61 ± 0.22	2.85 ± 0.92	0.178	0.296	0.922	0.273
TG/(mmol/L)	0.50 ± 0.20	0.50 ± 0.15	0.61 ± 0.10	0.57 ± 0.14	0.60 ± 0.14	0.031	0.741	0.420	0.576
TCHO/(mmol/L)	2.54 ± 0.17	2.16 ± 0.71	1.96 ± 0.45	1.88 ± 0.63	2.73 ± 0.25	0.123	0.117	0.201	0.081
UREA/(mmol/L)	4.93 ± 1.32	3.50 ± 0.97	4.74 ± 0.81	5.00 ± 0.35	5.23 ± 0.86	0.269	0.119	0.006	0.454
TP/(g/L)	65.11 ± 7.53	58.63 ± 10.37	60.27 ± 8.47	60.93 ± 7.95	75.10 ± 2.25	2.064	0.058	0.015	0.137
ALB/(g/L)	24.19 ± 1.88	22.19 ± 5.99	20.62 ± 1.90	20.58 ± 3.27	25.80 ± 2.50	0.833	0.195	0.224	0.096
GLB/(g/L)	40.93 ± 5.93	36.43 ± 6.44	39.65 ± 8.04	40.34 ± 4.75	49.30 ± 3.78	1.539	0.081	0.012	0.356
H-DLC/(mmol/L)	0.88 ± 0.07	0.84 ± 0.10	0.88 ± 0.09	0.86 ± 0.09	1.02 ± 0.17	0.050	0.209	0.093	0.256
L-DLC/(mmol/L)	1.11 ± 0.16^a^	0.93 ± 0.22^ab^	0.74 ± 0.16^b^	0.70 ± 0.18^b^	0.88 ± 0.17^ab^	0.048	0.040	0.438	0.095
ALP/(U/L)	74.53 ± 5.75	79.14 ± 14.86	70.22 ± 9.62	72.10 ± 4.76	84.96 ± 9.37	2.255	0.242	0.417	0.056
AST/(U/L)	20.75 ± 3.75	18.93 ± 3.64	17.52 ± 1.79	18.02 ± 1.65	21.15 ± 5.28	0.770	0.514	0.368	0.212
ALT/(U/L)	55.65 ± 10.51	42.75 ± 11.37	41.02 ± 7.58	41.87 ± 6.49	53.10 ± 8.87	2.307	0.107	0.130	0.166

### 3.3 Effect of dietary LCO levels on serum antioxidant indices in Taoyuan black pigs

As shown in Table 5, compared with the control treatment and *CTC* addition treatment of the basic diet, the catalase level in the serum of the pigs treated with 500 mg/kg LCO in the diet of finishing pigs was significantly increased (*p* < 0.05), and there was a significant secondary correlation between the catalase level in the serum of the pigs treated with different LCO levels in the diet (*p* < 0.05). In addition, T-AOC and the levels of SOD, GSH-Px, GSH, and MDA in serum of pigs were not significantly affected (*p* > 0.05).

**TABLE 5:** Effect of dietary LCO levels on serum antioxidant indices in *Taoyuan* black pigs.

Item	CTC level, mg/kg	LCO levels, mg/kg				SEM	*p*-value	*p*-value	
	75	0	250	500	1000			Linear	Quadratic
SOD/(U/mL)	35.19 ± 16.77	41.69 ± 10.11	39.07 ± 8.81	37.96 ± 10.35	39.72 ± 8.35	2.298	0.945	0.745	0.651
GSH-Px/(U/mL)	1751 ± 343	1553 ± 239	1737 ± 291	1656 ± 147	1626 ± 263	56.18	0.820	0.665	0.379
CAT/(U/mL)	4.19 ± 1.07^b^	4.65 ± 1.35^b^	5.61 ± 0.59^ab^	6.79 ± 1.08^a^	4.70 ± 0.34^b^	0.286	0.012	0.534	0.006
GSH/(µmol/L)	43.37 ± 11.71	37.70 ± 9.95	49.59 ± 3.65	53.23 ± 2.73	45.39 ± 5.10	2.440	0.087	0.029	0.017
T-AOC/(mmol/L)	1.17 ± 0.13	1.31 ± 0.05	1.13 ± 0.16	1.23 ± 0.22	1.29 ± 0.20	0.040	0.519	0.835	0.160
MDA/(nmol/mL)	2.55 ± 0.86	2.58 ± 0.58	2.44 ± 0.84	2.48 ± 0.61	2.23 ± 0.48	0.171	0.955	0.505	0.831

### 3.4 Effect of dietary LCO levels on serum immune indices in Taoyuan black pigs

The effects of different dietary levels of LCO on the serum immune-related indicators of pigs are shown in Table 6. There was no significant difference in the serum IgA, IgG, IgM, C3, and C4 levels of the test pigs between the treatments (*p* > 0.05), but from the numerical point of view, the diets added different levels of LCO increased serum immunoglobulin levels in experimental pigs.

**TABLE 6:** Effect of dietary LCO levels on serum immune indices in *Taoyuan* black pigs.

Item	CTC level, mg/kg	LCO levels, mg/kg				SEM	*p*-value	*p*-value	
	75	0	250	500	1000			Linear	Quadratic
IgA (µg/mL)	641.90 ± 36.78	634.34 ± 17.11	643.09 ± 39.82	647.85 ± 48.28	651.09 ± 54.70	8.923	0.982	0.564	0.980
IgG (mg/mL)	19.60 ± 1.73	17.24 ± 1.63	19.71 ± 2.14	18.77 ± 1.26	18.57 ± 1.73	0.405	0.304	0.284	0.146
IgM (mg/mL)	19.70 ± 0.72	19.93 ± 1.39	20.15 ± 1.02	19.62 ± 0.79	20.55 ± 1.66	0.310	0.799	0.681	0.631
C3 (µg/mL)	107.26 ± 12.24	103.37 ± 7.09	101.94 ± 7.74	104.78 ± 4.04	100.02 ± 9.07	1.965	0.789	0.685	0.700
C4 (µg/mL)	7.62 ± 0.85	7.62 ± 0.70	7.16 ± 0.45	6.94 ± 0.66	7.47 ± 0.85	0.194	0.605	0.529	0.234

### 3.5 Effect of dietary LCO levels on nutrient apparent digestibility in Taoyuan black pigs

Table 7 shows the effects of different dietary levels of LCO on the apparent digestibility of nutrients in pigs. Compared with the control treatment fed the basal diet and the *CTC* treatment, the experimental treatments with different levels of LCO increased the apparent digestibility of DM and GE and decreased the apparent digestibility of CF (0.05 < *p* < 0.10). Moreover, the apparent digestibility of CP, Ash, and Ca in pigs with different levels of LCO was significantly increased compared with the control treatments fed the basal diet (*p* < 0.05). In addition, compared with the control treatment fed the basal diet and the treatment with *CTC*, the apparent digestibility of EE in pigs treated with medium-dose LCO was significantly increased (*p* < 0.05), and the apparent digestibility of pigs was significantly increased after the addition of low-dose LCO (*p* < 0.05). At the same time, the apparent digestibility of CP, Ash, and Ca, as well as the changing trend of the ADE, had noticeable linear positive correlations with different dietary levels of LCO (*p* < 0.05). These indicated that the addition of different levels of LCO in the diet could significantly improve the efficiency of nutrient digestion and absorption in pigs.

**TABLE 7:** Effect of dietary LCO levels on nutrient apparent digestibility in *Taoyuan* black pigs.

Item	CTC level, mg/kg	LCO levels, mg/kg				SEM	*p*-value	*p*-value	
	75	0	250	500	1000			Linear	Quadratic
DM	84.09 ± 0.57	82.18 ± 1.14	84.91 ± 2.76	85.58 ± 3.20	85.66 ± 2.20	0.464	0.065	0.017	0.439
CP	82.03 ± 1.08 ab	80.49 ± 1.22b	82.84 ± 1.09a	83.25 ± 2.05a	83.16 ± 1.36a	0.336	0.021	0.004	0.218
EE	87.29 ± 1.02b	87.84 ± 1.34b	88.48 ± 0.79 ab	89.83 ± 0.53a	88.63 ± 0.92 ab	0.258	0.013	0.051	0.242
Ash	34.95 ± 2.05a	30.51 ± 1.88b	36.10 ± 2.82a	37.28 ± 3.93a	36.64 ± 3.37a	0.739	0.012	0.004	0.141
CF	44.95 ± 5.51	38.20 ± 6.48	35.45 ± 3.79	39.11 ± 3.33	36.23 ± 2.84	1.187	0.056	0.724	0.862
Ca	39.99 ± 2.76 ab	34.33 ± 3.47b	41.63 ± 6.39a	43.10 ± 4.28a	43.98 ± 4.36a	1.082	0.020	0.005	0.379
P	48.41 ± 3.81	46.89 ± 2.84	44.16 ± 3.13	44.90 ± 3.19	49.17 ± 4.87	0.766	0.224	0.665	0.048
GE	86.38 ± 0.68	85.46 ± 1.23	88.62 ± 1.99	88.03 ± 2.66	87.49 ± 2.25	0.404	0.067	0.066	0.098
ADE (Mcal/kg)	3.27 ± 0.03b	3.17 ± 0.06c	3.38 ± 0.11a	3.25 ± 0.04bc	3.27 ± 0.03b	0.018	0.001	0.023	0.005

### 3.6 Effect of dietary LCO levels on bacterial diversity in the feces of Taoyuan black pigs

**FIGURE 1:**
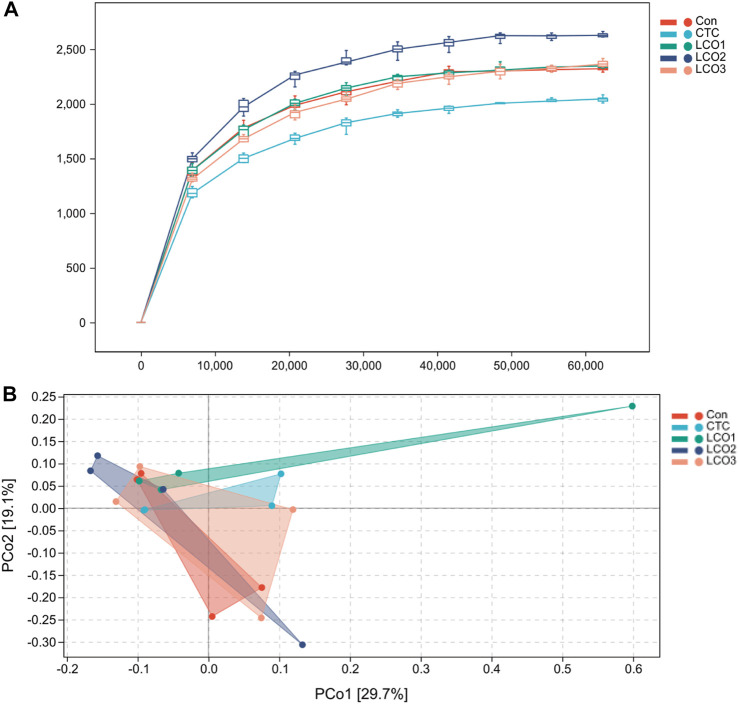
**(A)** Rarefaction curves of observed bacterial sequences in the fecal contents of pigs. **(B)** Principal coordinates analysis (PCoA) of fecal contents bacterial community of pigs. Con represents the group fed a basal diet, and CTC represents the group fed a basal diet with antibiotics, LCO1, LCO2, and LCO3 combined with 250 mg/kg, 500 mg/kg, and 1000 mg/kg, respectively.

The rarefaction curves reached a plateau and fully measured most of the bacterial diversity (Figure 1A). In addition, the PCoA showed that the *CTC* group stayed away from the LCO1 group, and the other group sets of samples were not completely separated (Figure 1B).

**FIGURE 2:**
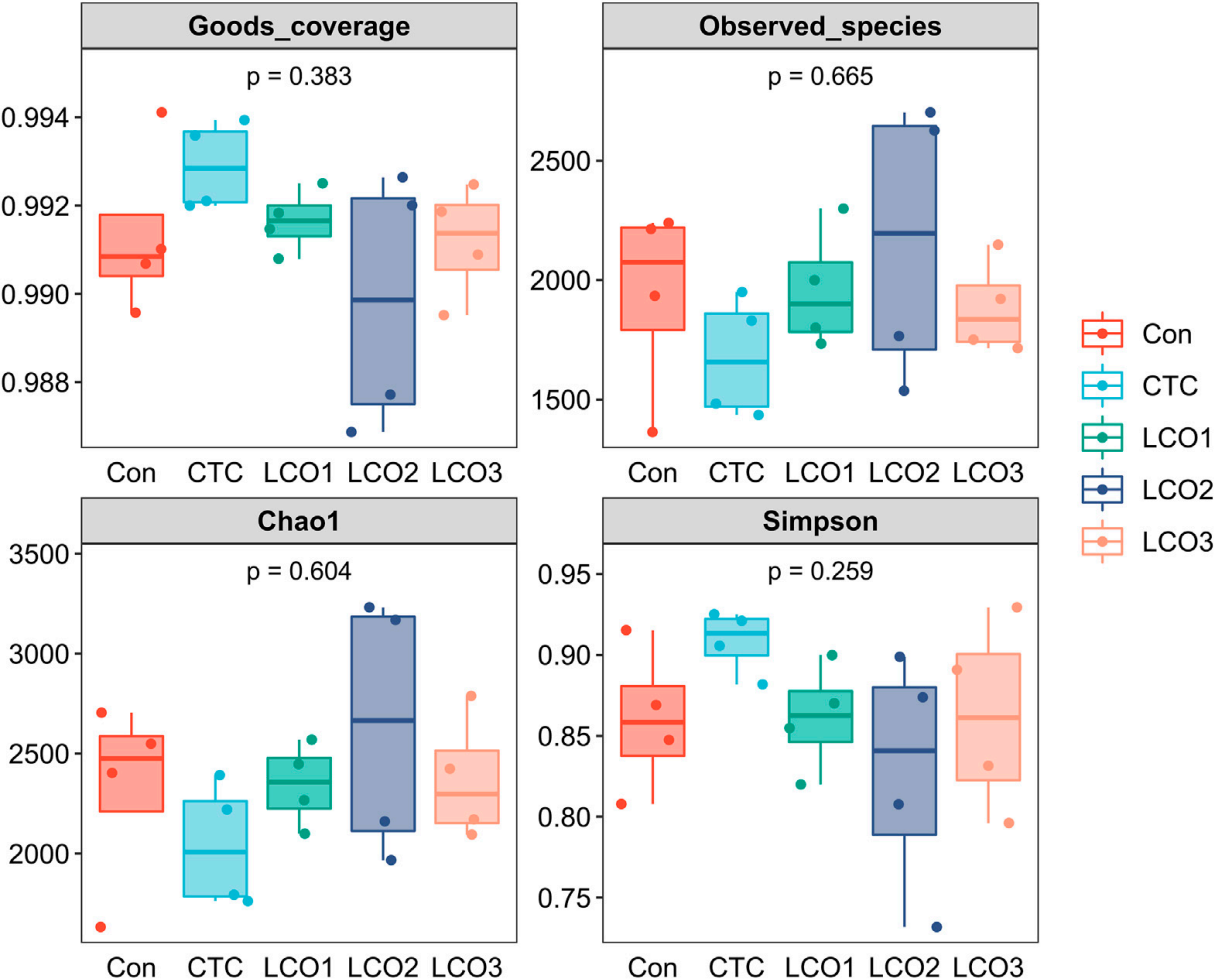
Alpha diversity indices of the fecal bacterial communities in pigs. Con represents the group fed a basal diet, and CTC represents the group fed a basal diet with antibiotics, LCO1, LCO2, and LCO3 combined with 250 mg/kg, 500 mg/kg, and 1000 mg/kg, respectively.

Based on the five treatments, Figure 2 shows the differences in fecal bacterial diversity among *Taoyuan* black pigs. The bacterial composition of the LCO2 group had a higher Chao1 estimator and lower Simpson index than the other four groups, and the *CTC* group showed the lowest observed_species and Chao1 in the feces. However, the difference between the four indicators in the five groups was not statistically significant (*p* > 0.05).

**FIGURE 3:**
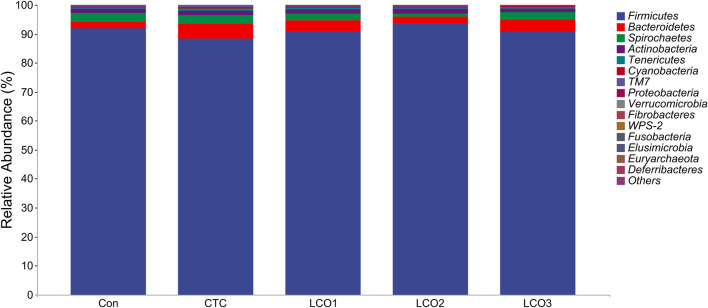
Distribution of fecal bacteria at the phylum level in pigs. Con represents the group fed a basal diet, and CTC represents the group fed a basal diet with antibiotics, LCO1, LCO2, and LCO3 combined with 250 mg/kg, 500 mg/kg, and 1000 mg/kg, respectively.

**FIGURE 4:**
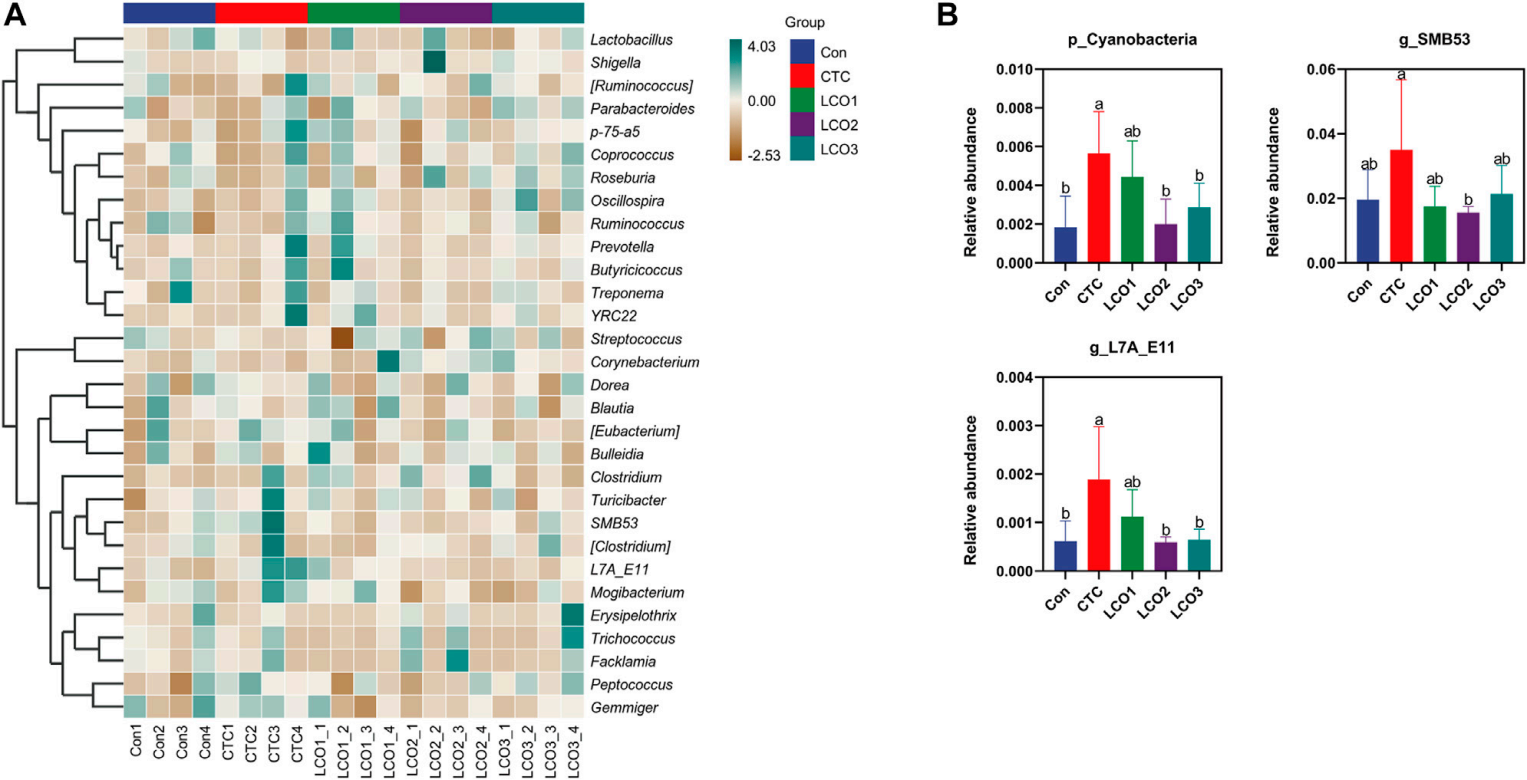
Effects of different concentrations LCO on fecal bacteria at the genus level in pigs. **(A)** Distribution of fecal top 30 bacteria at the genus level in pigs. **(B)** Distribution of different bacteria at the genus level in pigs. Con represents the group fed a basal diet, and CTC represents the group fed a basal diet with antibiotics, LCO1, LCO2, and LCO3 combined with 250 mg/kg, 500 mg/kg, and 1000 mg/kg, respectively.

*Firmicutes*, *Bacteroidetes*, and *Spirochete*s were the dominant phyla in the feces of *Taoyuan black* pigs, accounting for more than 90% of the total fecal bacterial community (Figure 3). When the diet of LCO proportion increased from 0% to 0.1%, the abundance of cyanobacteria in Con, LCO2, and LCO3 was significantly decreased than that in the *CTC* group (*p <* 0.05). Within the bacterial population, the top 30 genera were identified across all samples (Figure 4A), and the genus *Streptococcus* was the most abundant genera, followed by *Lactobacillus*, *Treponema*, *SMB53*, and *Clostridium*, which were predominant genera of feces in the *Taoyuan black* pigs. Among the genera, the percentage abundance of *SMB53* (3.50 ± 2.17 vs. 1.56 ± 0.19; *p <* 0.05) was decreased in the feces of the *CTC* group when compared to that in the LCO2 group (Figure 4B). At the same time, the relative abundance of *L7A_E11* was markedly decreased in the feces of the Con, LCO2, and LCO3 group than that in the *CTC* group (*p <* 0.05).

## 4 Discussion

Currently, traditional Chinese medicine (TCM) research mainly focuses on polysaccharides and flavonoids (Zhong et al., 2016; Ma et al., 2021). There have been many studies on the application effect of plant essential oil in piglet production, but the results vary. The study of Li SY. et al. (2012) showed that the addition of 100 or 150 mg/kg plant essential oils containing thymol and cinnamaldehyde to the diet of weaned piglets could significantly improve the average daily gain and feed conversion efficiency. The study of Gois et al. (2016) showed that the growth performance of piglets was unaffected by pepper essential oil, but it increased the density of intestinal villi. Charal et al. (2016) added 50 mg/kg star anise essential oil to the diets of lactating sows and weaned piglets. There was no significant difference between star anise essential oil and other essential oils in terms of affecting sow performance, but weaned piglets consumed significantly more feed after being fed star anise essential oil. The results of this experiment showed that adding different levels of LCO to the diets of finishing pigs did not significantly affect their growth rates, but the treatment group adding 250 mg/kg LCO in the diet obtained the best feed weight ratio. This indicated that adding an appropriate amount of LCO to the diet for fattening pigs can create economic benefits by saving feed.

In addition to providing information about the body’s health and immune function, blood biochemical indexes reveal its biological characteristics (Hussain et al., 2016). Animal growth performance is affected by oxidative stress (Mruk et al., 2002), and due to excess production and accumulation of reactive oxygen species (ROS), this imbalance occurs in the oxidative system (Hussain et al., 2016). MDA is a metabolite produced by peroxidation between free radicals and biofilm lipids. MDA reflects the extent of tissue peroxidation and indirectly indicates cell damage caused by oxygen free radicals. SOD is a common antioxidant enzyme in the animal body, which can catalyze the disproportionation of superoxide anion (O^−^_2_) into oxygen (O_2_) and hydrogen peroxide (H_2_O_2_) and remove O^−^_2_ from the mitochondrial membrane and mitochondrial matrix, which is usually an indicator of the body’s ability to respond to oxidative stress. GSH-Px is involved in the reduction of glutathione. T-AOC is used to reflect the scavenging ability of the antioxidant system to oxygen free radicals, and it is often used as a reference index of total antioxidant capacity (Moine et al., 2018; Li et al., 2019). Previous studies have shown that the addition of oregano essential oil (Zhang et al., 2015), cinnamaldehyde (Luo et al., 2020), and the mixture of cinnamaldehyde and thymol (Tian and Piao, 2019) can increase the activity of antioxidant enzymes and reduce the content of MDA in serum. This experiment proved that LCO can improve the level of CAT in the body to improve the ability of pigs to cope with stress. IgA, IgG, and IgM are the main substances of animal humoral immunity, and they can specifically combine with corresponding antigens to play a role. Serum IL-1 β, IL-10, and IFN-γ are involved in the immune response of many kinds of cells, and their contents can judge the inflammation of the body. The content of immunoglobulin can be used as an index to evaluate the immune function of weaned piglets (Grivennikov et al., 2010). Although LCO has no significant effect on the content of IgA, IgG, and IgM in pig serum, from the numerical point of view, the addition of different levels of LCO to the diet has increased the level of pig serum immunoglobulin to a certain extent.

Studies have shown that plant essential oils can increase the secretion and activity of endogenous digestive enzymes by increasing the secretion of animal saliva and bile and finally improve the digestibility of nutrients in the diet (Lee et al., 2003). The study of Zeng et al. (Yan et al., 2010) also showed that the addition of plant essential oils to the diet could significantly improve the apparent digestibility of crude protein in piglets. Amad et al. (2011) demonstrated that the addition of mixed essential oils of star anise and thyme could significantly improve the apparent digestibility of crude protein and crude fat in broilers. This experiment was conducted to explore the effect of LCO on nutrient digestibility of *Taoyuan black* pigs in order to clarify the effect of LCO on nutrient digestion of pigs. Based on the results, the addition of LCO could effectively improve the apparent digestibility of EE in weaned pigs. This may be due to the fact that the fat in the diet breaks down into free fatty acids and glycerol under the action of intestinal lipase and enters the bloodstream in the form of chylous particles (Qing-Hui et al., 2016). An imbalance in the interaction between gut microbiota and other factors can disrupt intestinal mucosal homeostasis (Luo et al., 2022). Studies have shown that the addition of plant essential oils to animal diets is beneficial to the balance of intestinal microorganisms and the secretion of digestive enzymes (Helander et al., 1998; Jang et al., 2004). Zeng et al. (2015b) significantly increased the digestibility of dry matter, crude protein, and energy after adding cinnamaldehyde and thymol to the diet of piglets. The experiment of Yan et al. (2010) in growing and finishing pigs demonstrated that the addition of menthol-based plant essential oil significantly improved the digestibility of crude protein and main amino acids, while the addition of cinnamaldehyde-based plant essential oil did not significantly affect the apparent digestibility of dietary nutrients. Li P. et al. (2012) found that the addition of 18 mg/kg thymol and cinnamaldehyde to the diet of weaned piglets could increase the digestibility of nutrients, reduce the content of IL-6 in blood and the number of *Escherichia coli* in feces, and increase the total antioxidant capacity in blood, but it did not significantly affect the growth performance of piglets. Van Noten et al. (2020) added 500 mg/kg thymol to the diet of weaned piglets and found that it decreased intestinal permeability and diarrhea rate, but had no significant effect on the growth performance of pigs. Thus, it can be seen that there is a significant difference in the addition level of essential oil in the diet of weaned piglets, and the adding effect is different, which needs to be further studied. Finally, the diet supplemented with different levels of LCO could significantly improve the nutrient digestion and absorption efficiency of finishing pigs.

It is very important to study fecal microbiota for the growth and health of animals (Wu et al., 2017; Wang H. et al., 2019; Wang et al., 2022c). Microbiota in the gut play an important role in digestion, metabolism, immunity, and pathogen defense in animals (Cani and Delzenne, 2009; Wang et al., 2021). To study the complex relationship between the host and microbiota, it was essential to better understand how host and microbes interact. The development of gene sequencing technology allows us to study how changes in the animal diet affect the structure and function of intestinal microorganisms (He B. et al., 2020; Wang et al., 2022d). Several studies have demonstrated the antibacterial activity of various essential oils (Si et al., 2006; Tu et al., 2018). The study on the antibacterial model of plant essential oil by Helander et al. (1998) showed that plant essential oil decomposes the bacterial cell membrane and releases the substances in the membrane from the cell to the external medium. Animal experiments demonstrated that added plant essential oil could increase the number of lactic acid bacteria in piglet feces and reduce the number of *E. coli* (Wei and Shibamoto, 2007). In this study, lactic acid bacteria and *E. coli* in pig feces were not affected by the level of LCO. To maintain digestive function and absorption of nutrients, the intestinal structures and microbiota must remain intact. It was believed that the intestinal bacterial community played a significant role in preserving intestinal function (Sczesnak et al., 2011; Chen J. et al., 2015). The ecosystem contained more than 100 trillion microorganisms, mostly bacteria (Collins et al., 2012). Several factors affect the composition and activity of the microbiota in the intestine, including age, environment, and diet. Diet was the most important factor (Fan et al., 2015; Sonnenburg et al., 2016; Ma et al., 2017). Dietary patterns could affect not only weight but also bone density (Chen Y. et al., 2015). In addition to colonization, gut microbiota-mediated immunity was also influenced by the diet (Saresella et al., 2017). In this study, the bacteria at the major phyla and genus levels of pig feces were not significantly changed. Species-based definitions of a healthy gut microbiome were difficult due to the high variability between and within species (Nguyen et al., 2015; Sender et al., 2016). Even so, gut microbiota and metabolites seem to remain relatively stable (Sender et al., 2016; McCoy et al., 2017). The results of the study of Wei et al. (2017) showed that supplementation of the mixture of thymol and carvol could reduce the relative abundance of *Enterococcus* and *E. coli* and increase the *Lactobacillus* abundance in the jejunum of weaned piglets. Manzanilla et al. (2004) added 15 and 30 mg/kg carvanol, cinnamaldehyde, and pepper essential oil to the diet could significantly increase the ratio of lactic acid bacteria to enterobacteria in the jejunal chyme of piglets. In this experiment, the cyanobacteria abundance had a downward trend in LCO compared to *CTC* treatment. According to the study of Ley et al. (2005), the cyanobacteria group has a logical intestine-associated branch. It is possible that this group is the descendant of ancestral non-photosynthetic cyanobacteria that have adapted to live in animal gastrointestinal tracts.

## 5 Conclusion

To sum up, adding a certain level of LCO to the diet of pigs could improve the growth performance and blood physiological and biochemical conditions of pigs, improve the antioxidant level of the pig body and the efficiency of digestion and absorption of nutrients, and show the potential to replace antibiotics (CTC). In general, it was considered that 250 mg/kg of LCO should be added to the diet under the experimental conditions.

## Data Availability

The data presented in the study are deposited in the NCBI repository, accession number PRJNA953808.
